# Surgical avulsion of the nail plate as therapy for resistant onychomycosis: case series and literature review^☆^

**DOI:** 10.1016/j.abd.2024.06.002

**Published:** 2024-10-18

**Authors:** José Antônio Jabur da Cunha, Fernanda Santana Barbosa, Gustavo de Sá Menezes Carvalho, John Verrinder Veasey

**Affiliations:** a Dermatology Clinic, Hospital da Santa Casa de São Paulo, São Paulo, SP, Brazil; b Discipline of Dermatology, Faculdade de Ciências Médicas, Santa Casa de São Paulo, São Paulo, SP, Brazil

Dear Editor,

Onychomycosis is a fungal infection of the nails caused by dermatophytes, non-dermatophytic filamentous fungi (NDFF), and yeasts, and is the most commonly observed nail disease in clinical practice.^1^, ^2^ Although common, treatment can be challenging, given the rise in reported resistant cases, either due to an increase in the number of cases related to NDFF that have recognized resistance to classical treatment,^1^, ^2^ or due to virulence factors related to pathogens such as biofilm production, or due to the host's immunological inability to defend himself.^3^, ^4^

Contrary to what was previously thought, the fungi involved in onychomycosis are capable of alternating between the planktonic form and the biofilm presentations.^4^ The term planktonic refers to isolated fungal cells, freely suspended in a medium, whereas in the form of biofilms, these cells adhere to a surface and form extensive collaborative multicellular communities surrounded by an extracellular matrix.^4^, ^5^ In fact, despite being recognized as susceptible to antifungal drugs, the reasons why onychomycosis tends to be refractory to treatments are still uncertain, with a possible relationship with the microenvironment of the nail apparatus and the formation of biofilms suggested as possibilities.^5^

Ideally, biofilms should be removed before starting drug treatment, implying the need for combination therapies.^5^ Procedures such as onychoabrasion, laser, photodynamic therapy, chemical or surgical avulsion are some of the techniques suggested for removing/rupturing the biofilm, thus facilitating drug action.^4^, ^6^

A retrospective study was conducted, analyzing patients treated at the Dermatology Clinic between January 2016 and December 2023 with onychomycosis refractory to classical drug treatment, who underwent surgical avulsion as alternative therapy. Cases with clinical and onychoscopic suspicion of onychomycosis, and with direct mycological examination (DME) and fungal culture positive were included. The combination of oral antifungals (terbinafine and/or itraconazole) with topical antifungals in nail polish formulation (ciclopirox olamine 5% or amorolfine 8%) was considered as the classical treatment and those who did not show any clinical improvement after one year of pharmacological treatment were considered to be resistant.

Eight patients with 12 treated nails were included in the study (Table 1). All had DME with evidence of fungi; the etiological agent was isolated as NDFF in nine cases, *Neoscytalydium dimidiatum var. hyalinum* in six, *Fusarium sp* in three, in addition to one with *Trichophyton rubrum* and another with *Candida sp*. (Table 2). Regarding nail surgery, in eight cases it was performed via the proximal approach of the nail plate and in the other four via the distal approach. The distal technique was indicated for patients with evident onycholysis where nail detachment can be performed in a less traumatic manner. None of the patients developed complications in the postoperative period and all had nail plate growth after the procedure (Table 3). Because this is a procedure with low morbidity (with the use of local anesthesia) and short surgical time, none of the patients were excluded from the study.

**Table 1:** Clinical personal history of patients with refractory onychomycosis undergoing nail plate avulsion: gender, age and comorbidities.

Identification			
Number	Gender	Age	Comorbidities
1	Male	69	SAH, DLP
2	Male	69	SAH, DLP
3	Male	69	SAH, DLP
4	Female	37	None
5	Female	59	Hypothyroidism, fibromyalgia, osteoporosis
6	Male	58	SAH
7	Male	58	SAH
8	Female	67	SAH, iS, MCTD, panic syndrome
9	Female	44	None
10	Female	57	None
11	Female	57	None
12	Female	26	None

SAH, systemic arterial hypertension; DLP, dyslipidemia, iS, ischemic stroke; MCTD, mixed connective tissue disease.

**Table 2:** Characteristics of onychomycoses treated by nail avulsion: affected digit, results of mycological examinations and treatments performed prior to the procedure.

Identification	Preoperative			
Number	Affected digit	Direct Mycological Examination	Culture	Previous drug treatment
1	1 LT	Hyaline septate hyphae	*Neoscytalydium dimidiatum var hyallinum*^a^	ITRA (200 mg pulse) × TERB (500 mg pulse) + amorolfine nail polish (2×/week)
2	3 LT	Hyaline septate hyphae	*Neoscytalydium dimidiatum var hyallinum*^a^	ITRA (200 mg pulse) × TERB (500 mg pulse) + amorolfine nail polish (2×/week)
3	4 LT	Hyaline septate hyphae	*Neoscytalydium dimidiatum var hyallinum*^a^	ITRA (200 mg pulse) × TERB (500 mg pulse) + amorolfine nail polish (2×/week)
4	1 RT	Hyaline septate hyphae	*Trichophyton rubrum*	TERB (500 mg)
5	1 RT	Hyaline septate hyphae	*Neoscytalydium dimidiatum var hyallinum*^a^	ITRA (200 mg pulse) × TERB (5 mg pulse) + amorolfine nail polish (2×/week)
6	1 RT	Hyaline septate hyphae	*Fusarium sp.*^a^	TERB (5 mg pulse) + micolamina nail polish (2×/week)
7	1 LT	Hyaline septate hyphae	*Fusarium sp.*^a^	TERB (500 mg pulse) + micolamina nail polish (2×/week)
8	1 LT	Hyaline septate hyphae	*Candida sp.*	TERB (250 mg/d)
9	3 RF	Hyaline septate hyphae	*Fusarium sp.*^a^	ITRA (200 mg pulse) × TERB (500 mg pulse) + amorolfine nail polish (2×/week)
10	1 LT	Dematiaceous septate hyphae	*Neoscytalydium dimidiatum var hyallinum*^a^	ITRA (200 mg pulse) × TERB (500 mg pulse) + amorolfine nail polish (3×/week)
11	1 RT	Dematiaceous septate hyphae	*Neoscytalydium dimidiatum var hyallinum*^a^	ITRA (200 mg pulse) × TERB (500 mg pulse) + amorolfine nail polish (3×/week)
12	1 RT	Hyaline septate hyphae	Fusarium sp.^a^	ITRA (200 mg pulse) × TERB (500 mg pulse) + amorolfine nail polish (3×/week)

LT, left toe; RT, right toe; RF, right finger; ITRA, itraconazole; TERB, terbinafine.

a Agent isolated in at least three samples with an interval of at least two weeks between them, without isolation of any other pathogen.

**Table 3:** Evolution of patients after avulsion of the affected nail plate.

Identification	Surgical technique	Postoperative			
Number		Complication	Time of follow-up	Treatment	Evolution
1	Proximal	None	26 months	Micolamina nail polish 1 ×/w for 20 months	Healthy nail growth
2	Proximal	None	13 months	Micolamina nail polish 1×/w for 7 months	Healthy nail growth
3	Proximal	None	13 months	Micolamina nail polish 1×/w for 7 months	Healthy nail growth
4	Distal	None	15 months	TERB (500 mg pulse) + Butenafine cream 1×/d for 4 months	Healthy nail growth
5	Proximal	None	39 months	Micolamina nail polish 1×/w for 5 months	Dystrophic nail growth
6	Distal	None	31 months	TERB (500 mg pulse) isoconazole cream 1×/d + micolamina nail polish 1×/w for 15 months	Dystrophic nail growth
7	Proximal	None	28 months	TERB (500 mg pulse) + Butenafine cream 1×/d for 12 months	Dystrophic nail growth
8	Proximal	None	2 months	None	Healthy nail growth
9	Proximal	None	75 months	None	Healthy nail growth
10	Distal	None	62 months	TERB (500 mg pulse) + Tefin cream 1×/d for 5 months	Healthy nail growth
11	Distal	None	62 months	TERB (500 mg pulse) + Tefin cream 1×/d for 5 months	Healthy nail growth
12	Proximal	None	6 months	ITRA (200 mg pulse) + TERB (500 mg pulse) + micolamina nail polish 1×/w for 6 months	Healthy nail growth

TERB, terbinafine; ITRA, itraconazole; d, day; w, week.

Two surgical techniques were used: proximal total nail avulsion and distal total nail avulsion. Proximal digital blockade was used because the authors believe it is efficient and not very painful, with 2% xylocaine as a vasoconstrictor. After the digit was completely anesthetized, a tourniquet was applied. In the proximal technique, the nail plate is completely detached from the proximal nail fold using a nail remover tool, supporting the remover tool on the nail plate so as not to lose the correct plane. Once completely detached, the proximal portion of the nail is pulled above the proximal nail fold using a lever. This movement begins in the central portion of the nail and then extends to the lateral horns. Once the entire proximal portion of the nail plate has been retracted, it is gently detached from proximal to distal. In the distal technique, the nail plate is detached globally at all its connections with the nail folds, from distal to proximal, using a nail remover tool, and then the nail is removed. This technique tends to be more traumatic and it is not very useful when there is severe onychodystrophy.

After surgery, classic topical and oral antifungal treatment was maintained in all patients until the nail plate had grown back completely. The nine nails showed growth of the new plate without signs of infection during the follow-up period (Fig. 1). Three cases showed growth of the nail plate with dystrophy. After the avulsion only clinical signs were observed and no laboratory tests (DME or culture for fungi) were performed. The choice of using medication after the avulsion of the plate aimed at reducing pathogens in the nail apparatus (matrix, bed, folds) which could be a reservoir for recontamination, just as *tinea pedis* is in patients with recurrent onychomycosis.^1^ Several studies propose a combination of biofilm-targeted therapies and conventional drug treatments to increase the success rate in the treatment of refractory onychomycosis.^4^, ^6^, ^7^ Avulsion is a valid choice in this scenario, and according to the experience of the who conducted the study has a satisfactory cure rate.

**Fig. 1:**
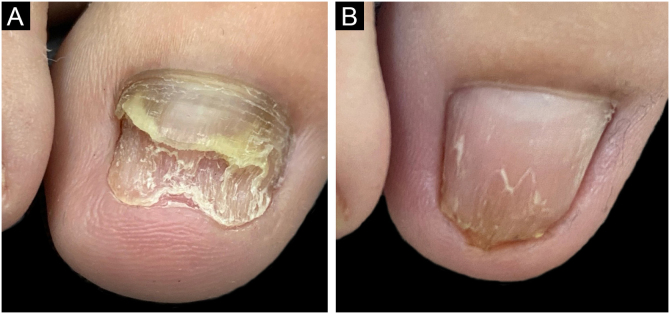
(A) Onychomycosis affecting the right hallux due to *Fusarium sp*. refractory to pharmacological treatment. (B) After six months of nail avulsion, followed by treatment with itraconazole (200 mg pulse), terbinafine (500 mg pulse) and micolamina nail polish.

Analyzing the cases in which the nail plate presented dystrophy after surgical avulsion, it is suggested that the condition may not be solely of infectious origin but may be associated with other factors such as nail trauma caused by patients walking habits.^6^, ^7^, ^8^ Given the considerable number of isolated NDFF, it is also possible to consider the hypothesis that these pathogens would not be the sole cause of the nail alteration, but rather secondary agents of onychomycotization. In these cases, it would be necessary to combine other therapeutic and behavioral measures in addition to fighting the infectious process to achieve complete improvement of the condition.^1^, ^4^, ^6^ It is important to emphasize that no patient developed an unfavorable condition after the procedure: in the worst scenario, the nail persisted with the previously observed dystrophy, with no case developing bleeding, infection, or pain.

The study data are promising and open perspectives for more robust studies, so it is important to discuss, at this time, avulsion as an alternative option for refractory cases, in association with systemic treatment. However, the study has limitations that need to be clarified: the small number of patients, lack of uniformity in the postoperative treatment, the fact that it is a retrospective study and no DME or culture was performed on the nails after the end of the treatment, especially in cases with dystrophy, which makes it difficult to prove the cure; in addition, there was no long-term follow-up, which hinders the assessment of therapeutic failure.

## Financial support

None declared.

## Authors' contributions

José Antônio Jabur da Cunha: Design and planning of the study; data collection, or analysis and interpretation of data; drafting and editing of the manuscript or critical review of important intellectual content; collection, analysis and interpretation of data; intellectual participation in the propaedeutic and/or therapeutic conduct of the studied cases; critical review of the literature; approval of the final version of the manuscript.

Fernanda Santana Barbosa: Data collection, or analysis and interpretation of data; statistical analysis; collection, analysis and interpretation of data; critical review of the literature.

Gustavo de Sá Menezes Carvalho: Data collection, or analysis and interpretation of data; statistical analysis; collection, analysis and interpretation of data; critical review of the literature.

John Verrinder Veasey: Design and planning of the study; data collection, or analysis and interpretation of data; drafting and editing of the manuscript or critical review of important intellectual content; collection, analysis and interpretation of data; intellectual participation in the propaedeutic and/or therapeutic conduct of the studied cases; critical review of the literature; approval of the final version of the manuscript.

## Conflicts of interest

None declared.
